# Effect of different culture systems on the production of foot and mouth disease trivalent vaccine

**DOI:** 10.14202/vetworld.2016.32-37

**Published:** 2016-01-12

**Authors:** Amr Ismail Hassan

**Affiliations:** Department of Foot and Mouth Disease, Veterinary Serum and Vaccine Research Institute, Abbasia, Cairo, Egypt

**Keywords:** baby hamster kidney-21 cell culture, foot and mouth disease virus, monolayer tissue culture cells, suspension tissue culture cells

## Abstract

**Aim:**

This study aims to determine the effect of the stationary rawx, roller, and the suspension cell culture systems on the total virus yield infectivity and antigenicity.

**Materials and Methods:**

Three serotypes of foot and mouth disease virus (FMDV) (serotype A, O and SAT-2) were inoculated separately into baby hamster kidney-21 cell line in rawx, roller, and suspension cultivation systems using multiplicity of infection (1:100). Samples were taken from the total virus yield from each system at 15, 18, 21, and 24 h post-inoculation. Testing the total virus yield infectivity through virus titration and antigenicity through estimation of complement fixing titer and 146S content and evaluation of the potency of the vaccine prepared from the different cultivation systems were done.

**Results:**

The results showed that the FMDV titer of serotype A, O, and SAT-2 obtained from the roller cultivation system showed the highest level followed by suspension cultivation system then the rawx cultivation system. The FMDV titer showed its highest level at 21 h post-inoculation in all the cultivation systems and then decline at 24 h post-inoculation. The antigenicity reached its highest value content at 18 h post-inoculation either by complement fixation test or by quantifying the 146S intact virion. Montanide ISA 206 oil inactivated trivalent vaccines were prepared from the tested serotypes (A Iran O5. O Panasia and SAT-2/EGY/2012) harvested at 18 h post-inoculation from the 3 culture systems. The results of tracing the antibody response showed that the mean antibody response from the roller cultivation system start its protective antibody titer earlier at 2 weeks post-vaccination (WPV) than the vaccine prepared from the other two cultivation system and the immune protection period lasts longer for 36 WPV for the roller cultivation system vaccine than the other two cultivation systems.

**Conclusion:**

The best cultivation system used for the production of FMD vaccine regarding its highest infectivity and antigenicity is the roller system.

## Introduction

Foot and mouth disease (FMD) is considered a highly infectious disease of cattle, sheep, goats, and pigs. It also affects wild animals such as buffaloes and deer [1,2]. It is characterized by rise in body temperature with saliva drooling from its mouth due to vesicular lesions on the tongue, gums, cheeks, and hard palate. The sick animals also show vesicular lesions in the cleft of feet, at their coronary bands and on teats of the milking animals. The vesicles soon rupture to form ulcerative lesions. There is a significant reduction in milk production, working efficiency and weight gain and abortions in infected animals. There is 100% morbidity in susceptible animal population and negligible mortality in adults. However, high mortality in a young animal is due to the virus-induced necrotic lesions and myocardial degeneration [3,4]. The etiologic agent of the disease is the FMD virus (FMDV) which has seven serotypes of FMDV have been recognized including O, A, C, SAT-1, SAT-2, SAT-3, and Asia 1 [2]. Although FMD has a low mortality figure, its high morbidity and contagiousness can lead to enormous economic consequences [5].

In Egypt, The type O was the most prevalent since 1960 [6-8]. Serotype A was introduced to Egypt in 2006 through importation of animals [9]. In addition, FMDV serotype SAT-2 of FMDV was introduced to Egypt in 2012 [10]. In Egypt and many other countries, the live animal’s importation is considered as the main risk factor in many old and new outbreaks of FMD. The effective vaccination of susceptible animals is considered to be the corner stone to eliminate the disease in endemic areas for controlling the FMD in animals [11].

The control of FMD by animal vaccination was effective in limiting the spread of FMD [12]. Such control is dependent on the potency, efficacy, and specificity of the produced vaccine which is related directly on the infectivity and antigenicity of the vaccinal FMDV serotypes used in the vaccine.

FMDV grow well on baby hamster kidney (BHK)-21 cell line enabling large-scale production of antigen with good antigenicity. It has also been reported that with subsequent cultivation in BHK-21 clone 13 cell line, the titer of FMDV increased. Furthermore, the susceptibility and infectivity titer of BHK-21 is higher than IBRS-2 and Madin-Darby bovine kidney cell lines, and thus it is used for vaccine production. The field isolates of FMDV could be passaged in BHK-21 clone 13 monolayer cell culture either in rawx or roller system, which showed a characteristic cytopathic effect (CPE) after adaptation to 5^th^ passages. Another way to virus propagation is the cell suspension as the BHK-21 is suspended in a media at which it is propagated. However, the cell culture system is laborious, time-consuming, and relatively low sensitive. It also requires careful handling of samples and a good biosafety measures [13].

Hence, This work was designed to compare between the effect of different cell culture systems including stationary system (rawx), roller culture systems and suspension system on the production of (FMDV) type A, O and SAT-2 so as to improve the potency of the FMD vaccine produced.

## Materials and Methods

### Ethical approval

The experiment was performed according to the protocol of Institutional Animal Ethics Committee, and the authors had taken permission of animal owners at the private farms.

### Animals

17 native breed cattle from a farm in Sharkia Governorate, Egypt were found to be serologically negative for the presence of antibodies against FMDV type A, O or SAT-2 as proved by SNT and ELISA. These animals were used to determine the potency of the prepared vaccines.

### Virus serotypes

FMDV serotypes (A IranO5, OPanAsia and SAT-2/Egypt 2012) were supplied by FMD research department, VSVRI were used and propagated twice in a primary monolayer of bovine kidney cells and then in BHK-21 monolayer cells through 6 serially passages.

### Stationary rawx and roller cell culture cultivation systems

BHK monolayer cell cultures (BHK-21) clone 13 was obtained from the World Reference Lab. Pirbright Surrey, U.K. The cells were serially passage and maintained in the FMD Research Department, Veterinary Serum and Vaccine Research Institute, Abbasia, Cairo [14]. These cells were grown in rawx flasks as stationary system and in roller bottle as roller culture system. Minimum Essential Medium (MEM) modified with Hank’s salt was supplied by Flow Laboratories, U.K. It was used as maintenance medium with 1-2% fetal calf serum added and was adjusted to pH 7.6-7.8 for the infected cell cultures at 37°C. Inoculation of rawx and roller systems were inoculated separately with FMDV serotypes A, O and SAT-2.

### Suspension cell culture cultivation system

BHK-21 clone 13 suspended cells from liquid nitrogen were suspended in complete growth media (especially contain fetal calf serum), where cell concentration was adjusted between 0.4 and 0.5×10^6^ cells/ml. The Bellco bottle (vessel) was incubated at 36.5°C and good cell growth was indicated by an increase of 100% or more in cell number after 24 h incubation period. After 48 h of incubation, the viable cell number reached 2.0×10^6^ cells/ml or more if exponential growth has been maintained. When the concentration of cells reached for example 2.0×10^6^ cells/ml or more the cells either can be diluted to be cultivated again or centrifuged and resuspended in maintenance media (Hanks media) for cultivation of virus (virus inoculation). The cell number was determined by counting in “Modified Fuchs-Rosenthal” by using 0.05% trypan blue as a vital stain [15]. Inoculation of bellco bottles were inoculated separately with FMDV serotypes A, O and SAT-2.

### Experimental design

The 6^th^ BHK-21 passage of type A IranO5, OPanAsia and SAT-2/Egypt were inoculated separately on BHK-21 cells in roller bottles, rawx bottles and suspension bellco bottles using Multiplicity of Infection (MOI) as 1:100 according to Community Coordinating Institute Netherlands. Samples were obtained at 15, 18, 21 and 24 h post-virus inoculation. Samples were collected and freezed and thawed for three times and then kept at −70°C (representing the total virus yield).

### FMDV purification

Aseptically, the harvested culture medium from each FMDV serotype infected BHK-21 cell cultures were centrifuged in a cooling centrifuge at 7000 rpm for 20 min to remove cell debris [16].

### FMDV serotypes concentration

The purified tissue culture viral fluids of each serotype were concentrated by PEG-6000 to reach 1/10 of its original volume [17].

### FMDV infectivity titration

Titration of the obtained virus samples were carried out and CPEs of the virus was observed under an inverted microscope and processed for biological titration (TCID50) [18].

### Complement fixation test (CFT)

It was carried out according to Health Protection Agency [19] to detect the virus antigenicity.

### Estimation of 146S content of the FMDV serotypes

The content of 146S particles in prepared viral antigen estimated by using sucrose density gradient ultracentrifugation by determining the absorbance at 254 nm using ISCO 520C density gradient system according to Bartelling *et al*. [20].

### Preparation of the trivalent FMD vaccine from the cultivation system with the highest antigenic virus yield

The trivalent FMD vaccine with Montanide ISA 206 were prepared according to Gamil [21] which are formed from the highest antigenicity virus serotypes harvested at 18 h post-inoculation from the different cell culture cultivation systems from the previously tested serotypes of O pan Asia-2, A Iran O5 and SAT-2/Egy/2012. Each of 5 cattle was inoculated as 3 ml for each vaccine and Serum samples were collected pre-vaccination and every week until 4^th^ week, every 2 weeks for 16^th^ week, every 4 weeks until 32^nd^ week, and lastly, every 2 weeks until 42^nd^ week. Serum neutralization test was performed on the serum samples to detect the antibodies against serotypes O, A and SAT-2 as described by Ferreira [22] and expressed as log_10_. *Two cattle were left as a control negative along the experiment*.

## Results and Discussion

The main scope in vaccine production procedures is usually constructed in the production of a large amount of vaccine doses so as to fulfill the huge need of vaccine doses for different animals and also in addition to the potency of the produced vaccine regarding its high infectivity and antigenicity of the serotypes included in the vaccine.

As the BHK-21 cell line either the monolayer or the suspension systems are used in FMD vaccine production and to ensure the potency and the quality of the vaccine produced, It was of interest to follow-up the infectivity, complement fixing activity and 146S content of FMDV type A, O and SAT-2/Egypt 2012 on BHK cell culture in roller, rawx and suspension culture systems. The FMDV serotypes A, O and SAT-2 were inoculated with an MOI (1:100).

The FMDV is characterized by its short eclipse phase as the virus can be detected after 3 h post-inoculation [23]. The virus increases in growth and multiplication on the BHK-21 and subsequently increases in its infectivity and antigenicity. In this study, the infectivity and antigenicity of the virus yield were examined at 15, 18, 21 and 24 h post-inoculation.

**Figure-1:**
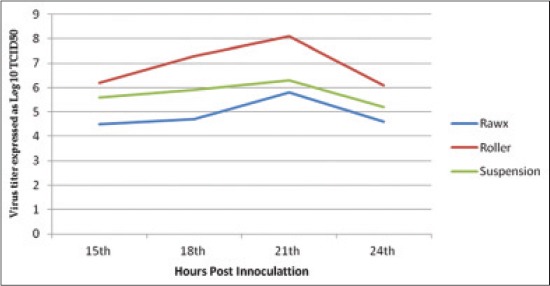
Effect of difference cell culture systems on foot and mouth disease virus serotype (A) infectivity in baby hamster kidney-21 cell line at 37°C.

**Figure-2:**
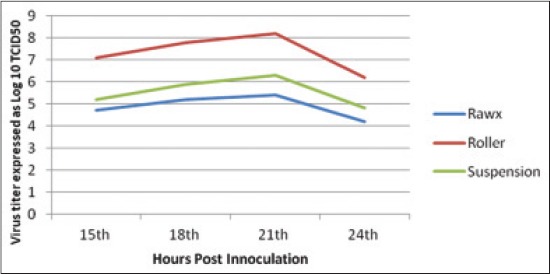
Effect of different cell culture systems on foot and mouth disease virus serotype (O) infectivity in baby hamster kidney-21 cell line at 37°C.

**Figure-3:**
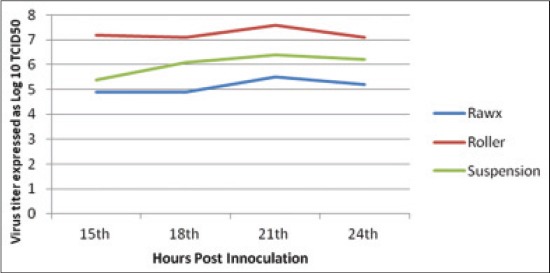
Effect of different cell culture systems on foot and mouth disease virus serotype (SAT-2) infectivity in baby hamster kidney-21 cell line at 37°C.

The virus titer yield from the different culture systems of cultivation including roller, rawx and suspension culture systems for serotype A, O and SAT-2 were detected at 15, 18, 21 and 24 h post-inoculation as Figures-1-3, the results showed that there is difference in the titer between the different cultivation systems at all tested hours as the FMDV titer type A, O and SAT-2 obtained from the roller cultivation system showed the highest level followed by suspension cultivation system then the rawx cultivation system. The FMDV titer showed its highest level at 21 h post-inoculation in all the cultivation systems as at 21 h post-inoculation the FMDV titer was 8.1, 8.2, and 7.6 Log_10_ TCID_50_, respectively, for the roller system; as 6.3, 6.3, and 6.4 Log_10_ TCID_50_, respectively, for the suspension system; and as 5.8, 5.4, and 5.5 Log_10_ TCID_50_, respectively, for the rawx cultivation system for the serotypes A, O, and SAT-2, respectively, and then decline at 24 h post-inoculation as the FMDV titer was 6.1, 6.2, and 7.1 Log_10_ TCID_50_, respectively, for the roller system; as 5.2, 4.8, and 6.2 Log_10_ TCID_50_, respectively, for the suspension system; and as 4.6, 4.2, and 5.2 Log_10_ TCID_50_, respectively, for the rawx cultivation system for the serotypes A, O, and SAT-2, respectively. This finding is contributed to the result obtained by Ali *et al*. [23] who found that the highest infectivity for FMDV serotype A virus was achieved after 21 h post-inoculation. This difference in infectivity between the roller and rawx cultivation system may be attributed to the surface area difference between the rowx and roller cultivation systems as the rowx flasks has a 75 cm^2^ while the roller cultivation system has a surface area of 490 cm^2^ and subsequently the exposed area for growing cells is greater in the roller cultivation system and in agreement with Akram *et al*. [24] who found that the virus infectivity titer in the stationary monolayer of BHK-21 cells in roller flask is higher than that in the rawx flasks and also agreed with Altaf *et al*. [25], Khawaja *et al*. [26], and Salivac *et al*. [27] who stated that the infectivity titer of the virus is directly proportional to number of BHK-21 cells in the culture system.

**Figure-4:**
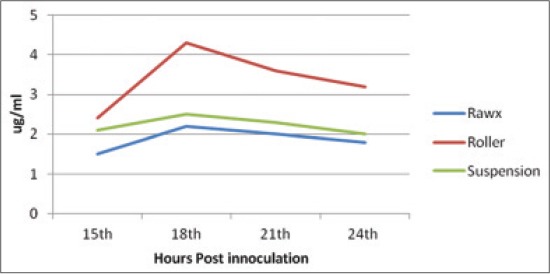
Effect of cell culture system on foot and mouth disease virus serotype (A) antigenicity (146S content) in baby hamster kidney-21 cell culture at 37°C.

**Figure-5:**
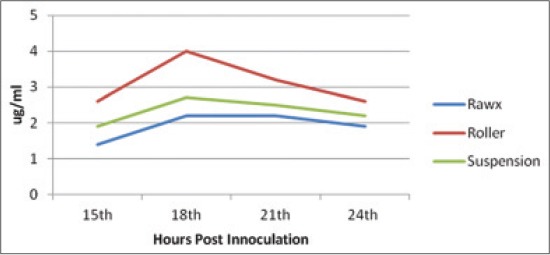
Effect of cell culture system on foot and mouth disease virus serotype (O) antigenicity (146S content) in baby hamster kidney-21 cell culture at 37°C.

**Figure-6:**
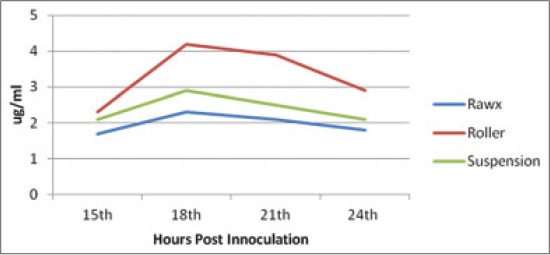
Effect of cell culture systems on foot and mouth disease virus serotype (SAT-2) antigenicity (146S content) in baby hamster kidney-21 cell line at 37°C.

Depending on the fact reported by Ali *et al*. [23] that the protective capacity of FMD vaccine is the FMDV antigenicity; the FMDV yield from different cultivation systems must be tested and compared on their antigenicity by CFT and quantifying the 146S intact virion content. The antigenicity detected by complement-fixing antibody titer (Table-1) showed that the highest titer was achieved by the roller cultivation system followed by the suspension and then the rawx cultivation system along all the tested hours post-inoculation and the highest complement fixing titer was achieved at 18 h post-inoculation for serotype A, O and SAT-2 1/64, 1/32, 1/64 for the roller system, respectively, and 1/32, 1/16 and 1/16 for the suspension cultivation system for serotype A, O and SAT-2, respectively, and 1/8 for the rawx system for serotype A, O and SAT-2 This result come parallel with that obtained by quantifying the 146S intact virion content as it reached its highest intact virion content at 18 h post-inoculation (Figures-4-6). The 146S intact virion content of serotype A, O and SAT-2 increases as it showed 4.3 ug/ml, 4 ug/ml, and 4.2 ug/ml for the roller system, respectively, and 2.5 ug/ml, 2.7 ug/ml, and 2.9 ug/ml for the suspension system, respectively, and 2.2 ug/ml, 2.2 ug/ml, and 2.3 ug/ml for the rawx cultivation system, respectively.

**Table-1:** Complement fixing titers of FMD virus serotypes using different cell culture systems.

HPI	FMDV serotype (A)			FMDV serotype (O)			FMDV serotype (SAT-2)		
		
	Rawx	Roller	Suspension	Rawx	Roller	Suspension	Rawx	Roller	Suspension
15^th^	1/4	1/32	1/16	1/8	1/32	1/16	1/4	1/32	1/16
18^th^	1/8	1/64	1/32	1/8	1/32	1/16	1/8	1/64	1/16
21^th^	1/8	1/16	1/8	1/4	1/16	1/16	1/4	1/8	1/8
24^th^	1/4	1/8	1/8	1/4	1/8	1/8	1/4	1/4	1/8

HPI=Hour post inoculation, FMDV=Foot and mouth disease virus

Hence, from the previous results it was clear that the roller cultivation system showed the highest antigenicity (complement fixing antibody titer, 146S intact virion content) followed by the suspension and then the rawx cultivation system and the best time to harvest the virus is 18 h post-inoculation as it reached its highest level of antigenicity for the three cultivation systems. This comes in agreement with Ali [28] who found that 146S content reached its maximum level by 18 h post-infection.

For making a confirmation between the pervious obtained results of the total virus yield from the different cultivation systems and the immune-protective effect against FMD, 3 montanide ISA 206 oil inactivated trivalent vaccines were prepared from the tested serotypes (A Iran O5. O Panasia and SAT-2/EGY/2012) harvested at 18 h post-inoculation from the 3 culture systems. Table-2 showed the results of the mean antibody response come in a parallel manner with the previous results as the vaccine prepared from the roller cultivation system start its protective antibody titer at 2 weeks post-vaccination (WPV) as 1.62 against serotype A, 1.58 against serotype O and 1.68 against serotype SAT-2 as the protection titer for FMD is 1.5 Log _10_ for SNT [29] while the vaccine prepared from the suspension cultivation system start its protective antibody titer at 3 WPV as 1.53 against serotype A, 1.56 against serotype O and 1.6 against serotype SAT-2 and the vaccine prepared from the rawx cultivation system start its protective antibody titer at 3 WPV as 1.51 against serotype A, 1.57 against serotype O and 1.53 against serotype SAT-2. The immune protection period varies between the different vaccines as it lasts for 36 WPV for the roller cultivation system vaccine while it lasts for 24 WPV for the suspension cultivation vaccine and it lasts for 16 WPV for the rawx cultivation system vaccine.

**Table-2:** Tracing of FMD serum neutralizing antibody titer induced by the vaccines prepared by different culture systems.

Vaccine type	FMD serum neutralizing antibody titer (Log_10_/ml)								

	Rawx cultivation system vaccine			Roller cultivation system vaccine			Suspension cultivation system vaccine		
		
WPV	FMDV serotype A	FMDV serotype O	FMDV serotype SAT-2	FMDV serotype A	FMDV serotype O	FMDV serotype SAT-2	FMDV serotype A	FMDV serotype O	FMDV serotype SAT-2
0	0.25	0.35	0	0.506	0.4	0.51	0.546	0.6	0.76
1	0.9	0.76	0.42	1.196	0.926	1.02	1.296	1.22	1.09
2	1.23	1.34	0.96	1.62	1.58	1.68	1.43	1.39	1.31
3	1.51	1.57	1.53	1.69	1.7	1.8	1.53	1.56	1.6
4	1.62	1.65	1.69	1.79	1.75	1.96	1.74	1.68	1.78
6	1.69	1.71	1.8	1.81	1.8	2.13	1.89	1.76	1.89
8	1.75	1.83	1.9	1.89	1.92	2.35	2.06	1.87	2.15
10	1.82	1.92	1.96	2.25	2.4	2.49	2.18	1.96	2.21
12	1.79	1.86	1.72	2.6	2.69	2.56	2.25	2.13	2.38
14	1.61	1.72	1.61	2.73	2.82	2.67	2.1	2.04	2.1
16	1.52	1.6	1.58	2.54	2.7	2.47	1.8	1.86	1.86
20	1.43	1.43	1.47	2.25	2.56	2.3	1.69	1.63	1.7
24	1.36	1.31	1.4	2.15	2.3	2.19	1.53	1.57	1.62
28	0.96	1.2	1.31	1.93	2.15	2.05	1.35	1.48	1.36
32	0.42	0.35	1.2	1.82	1.93	1.94	1.23	1.3	1.25
34	ND	ND	0.59	1.68	1.71	1.83	1.06	1.23	1.06
36	ND	ND	ND	1.58	1.67	1.7	0.94	1.06	0.65
38	ND	ND	ND	1.35	1.3	1.48	ND	0.87	ND
40	ND	ND	ND	1.21	1.15	1.3	ND	ND	ND
42	ND	ND	ND	0.92	0.96	1.18	ND	ND	ND

WPV=Week post-vsaccination, FMD=Foot and mouth disease, ND=Not detected, FMDV=Foot and mouth disease virus

## Conclusion

It is clear that the best cultivation system used for production of FMD vaccine regarding its infectivity and antigenicity is the roller cultivation system as it induce the highest titer and antigenicity either in the complement fixing titer or the 146S content among the other two types and that is reflected on the antibody titer and the protection period in vaccinated cattle.

## Authors’ Contributions

Amr Ismail has prepared the three tissue culture systems; prepare and inoculate the three foot and mouth disease virus serotypes; detect the infectivity of the FMDV by virus titration and the antigenicity by CFT and quantifying the 146S content; inactivate the virus yield; formulate the different vaccine; carry out the SNT and ELISA tests for antibody detection against FMD; wrote the manuscript and follow up the steps of publication.
